# Distribution and trends in outpatient utilization of generic *versus* brand name psychopharmaceuticals during a ten-year period in Croatia

**DOI:** 10.1186/1472-6963-14-343

**Published:** 2014-08-15

**Authors:** Marina Polić-Vižintin, Danijela Štimac, Zvonimir Šostar, Ingrid Tripković

**Affiliations:** Dr Andrija Štampar Institute of Public Health, Vrhovčev vijenac 22, HR 10000 Zagreb, Croatia; School of Medicine, University of Zagreb, Zagreb, Croatia; Institute of Public Health of Split and Dalmatia County, Zagreb, Croatia

**Keywords:** Outpatient utilization, Psychopharmaceuticals, ATC/DDD methodology, Generics, Pharmacoeconomics, Croatia

## Abstract

**Background:**

Drug costs increasingly pose a burden upon the otherwise inadequate health care resources and rational drug utilization is an important segment of every national health policy. Optimal patient care should be the goal of rational pharmacotherapy, whereby the economic burden of treatment is just one of the elements to be considered on choosing appropriate therapy.

The aim of this study was to determine distribution and trends in the outpatient utilization of generic *versus* brand name psychopharmaceuticals and to evaluate the rationality of prescribing psychopharmaceuticals during a ten-year period.

**Methods:**

Using the World Health Organization Anatomical-Therapeutic-Chemical classification/Defined Daily Doses (ATC/DDD) methodology, the number of DDD was calculated from data collected from pharmacies on the number and size of drug packages. The ratio of generic and brand name drug costs served as an indicator on assessing the rationality of drug utilization.

**Results:**

Total cost for psychopharmaceuticals increased by 20.1%, more for brand name than for generic agents (32.7% *vs*. 7.4%). The highest share of generic psychopharmaceuticals as compared with brand name drugs according to DDD *per* 1000 inhabitants *per* day (DDD/1000/day) was in the group of psycholeptics (83.6% in 2001 *vs*. 82.2% in 2010), most in hypnotics and sedatives, and least in antipsychotics. The share of generic psychopharmaceuticals in total drug utilization according to financial indicators decreased by 9.6% and according to DDD/1000/day by 12%. The greatest decrease was in antidepressants, i.e. by 33.8% according to financial indicators and by 46% according to DDD/1000/day; and in antipsychotics by 30.9% according to DDD/1000/day, while showing an increase by 8.5% according to financial indicators. In the therapeutic subgroup of mood stabilizers, the share of generic drugs in total drug utilization declined by 32% according to DDD/1000/day, but increased by 25.1% according to financial indicators.

**Conclusions:**

The lack of uniform national guidelines and the still strong impact of pharmaceutical industry marketing continue favoring the rise in prescribing brand name antidepressants and antipsychotics. Depression, schizophrenia and bipolar diseases are complex diseases. As a result, specific measures are needed to encourage the prescribing of generic psychopharmaceuticals.

## Background

Drug costs increasingly pose a burden upon the otherwise inadequate health care resources and rational drug utilization is an important segment of every national health policy [1]. The rising drug cost is influenced by the increasing number of new drugs, which as a rule are more expensive [2, 3]. Another factor influencing the rising drug cost is the prolonged life expectancy, along with the ever growing share of chronic diseases in the cost of pharmacotherapy and treatment in general [4]. In addition, neither should the impact of drug manufacturers and distributors, the attitudes, knowledge and personal characteristics of physicians, patient demands and expectations, healthcare structure, and governmental regulatory institutions and measures be neglected.

Rational pharmacotherapy implies the right agent prescribed in the right dosage to the right patient for the required period at the lowest cost for the individual and the community [2, 3, 5]. Optimal patient care should be the goal of rational pharmacotherapy, whereby the economic burden of treatment is just one of the elements to be considered on choosing appropriate therapy [3, 5].

Many European Union (EU) countries have successfully rationalized drug consumption by stimulating prescribing generic drugs, which are less expensive and as efficacious as brand name ones [4, 6–9].

This is especially the case when physicians are being encouraged through a variety of measures to preferentially prescribe generics [4].

During the last decade, the tools of external price referencing (international price comparisons) and reference price systems (limitation of reimbursement for identical or similar medicines in a cluster) have been increasingly used in several EU Member States. External price referencing is a common pricing procedure applied in 22 EU Member States [8].

In an attempt to bring measures to reduce the excessive financial costs for drugs and to rationalize drug utilization in general, during the study period the Croatian healthcare policy makers have enacted a number of bylaws and acts primarily aimed at drug consumption rationalization through direct or indirect means. In 2001, a new Act on Health Insurance was enacted [2]. In the same year, the Croatian Institute of Health Insurance (CIHI) issued a Directive on the List of Drugs, based on the Act, which included all the drugs that could be prescribed or administered during treatment at healthcare institutions in the Republic of Croatia. The List also regulated the level of patient co-payment for particular drugs [2, 10]. In 2002, the Ministry of Health of the Republic of Croatia issued the Bylaw on the Conditions and Implementation of Supplementary Health Insurance [11]. In 2004, aiming at drug cost containment, the Bylaw Regulating Wholesales Drug Pricing Policy and Reporting on Wholesale Prices was enacted, on the model of other European countries. According to the Bylaw, the criteria for wholesale drug pricing were their comparative prices in other countries, the level of their wholesale comparative prices, pharmacoeconomic studies, and reference price [12]. Comparative wholesale drug price was based on the same drug (of identical generic entity and identical pharmaceutical formulation) wholesale price in Italy, France and Slovenia. If data on the respective drug prices in these countries were lacking, then the prices in Spain and Czech Republic were taken for comparison. Since 2009, in order to be included in the essential CIHI list of drugs, the wholesale prices of the patented drugs should be by at least 10% lower than the average prices of the brand name drugs in the mentioned countries. The drugs that are not patent protected anymore should be cheaper by at least 35%, whereas the prices of generic drugs should be by at least 30% lower than their average prices in Italy, France and Slovenia. Only brand name drugs that contain a completely new active ingredient and appear on the Croatian market for the first time can have the same price as in the comparison countries, whereas the price of a drug of the same unprotected name cannot exceed 90% of the price of the last identical generic added to the list [8, 13].

In 2006, CIHI introduced two drug lists: main essential list containing all essential drugs covered by compulsory health insurance and supplementary list of drugs in part covered from compulsory health insurance and in part by the patients [14–16].

The Bylaw on the Policy of Including Drugs in the CIHI Essential and Supplementary list of Drugs has improved the methodology of issuing recommendations for drug placement on the list. Mandatory reporting on all promotion costs and financial transactions between pharmaceutical industry and physicians working in public health establishments has also been introduced [16].

Therefore, the aim of the present study was to determine distribution and trends in outpatient utilization of generic *versus* brand name psychopharmaceuticals in the City of Zagreb during the 2001–2010 period.

## Methods

In this retrospective, descriptive research, data on the number of drug packages and buying price for all prescription drugs registered in the Republic of Croatia were collected from all Zagreb pharmacies during the 2001–2010 period.

In order to make the data standardized and comparable with other settings, the World Health Organization Anatomical-Therapeutic-Chemical classification/Defined Daily Doses (ATC/DDD) methodology was used on assessing drug utilization [17].

The ratio of generic and brand name psychopharmaceutical costs served as an indicator on assessing the rationality of drug utilization [18]. The ratio of generic to brand name psychopharmaceutical costs was calculated by the following formulas:


### Ratio generic drugs/brand name drugs

Descriptive analysis was conducted for quantitative variables. Excel was used to create the database and to analyze the data. Data on drug utilization were handled in compliance with Zagreb Municipal Pharmacy.

### Ethics

This study was approved by the Ethics Committee of Dr Andrija Štampar Institute of Public Health. The research was in compliance with the Helsinki Declaration.

## Results

Outpatient utilization of psychopharmaceuticals (ATC subgroup N03-N06) in the City of Zagreb in 2001 and 2010, expressed according to financial indicators and DDD *per* 1000 inhabitants *per* day (DDD/1000/day), is illustrated in Tables 1, 2, 3, 4, 5, 6, 7, 8 and 9.

**Table 1:** **Outpatient utilization of psychopharmaceuticals (ATC subgroup N03-N06) in the City of Zagreb in 2001 according to financial indicators (€)**

ATC code	Therapeutic subgroup	Generic drugs (€)	Brand name drugs (€)	Total (€)	Share of generics%,	Share of brand name drugs%
N03AE-AX	Antiepileptics	116 101.72	138 313.4	254 415.12	45.63	54.37
N04 AA	Anticholinergics (biperiden)	0	38 566.1	38 566.10	0	100.00
N05	Psycholeptics	751 795.65	693 849.9	1 445 645.55	52.00	48.00
N06A	Antidepressants	173 367.48	189 463.97	362 831.45	47.78	52.22
Total		1 041 264.87	1 060 193.38	2 101 458.25	49.55	50.45

**Table 2:** **Outpatient utilization of psychopharmaceuticals (ATC subgroup N03-N06) in the City of Zagreb in 2001 according to DDD/1000/day**

ATC code	Therapeutic subgroup	Generic drugs DDD/1000/day	Brand name DDD/1000/day	Total DDD/1000/day	Share of generics% DDD/1000/day	Share of brand name drugs% DDD/1000/day
N03AE-AX	Antiepileptics	3.82	1.24	5.06	75.49	24.51
N04 AA	Anticholinergics (biperiden)	0	2.05	2.05	0	100.00
N05	Psycholeptics	78.44	15.38	93.82	83.60	16.40
N06A	Antidepressants	7.98	2.83	10.81	73.82	26.18
Total		90.24	21.5	111.74	80.76	19.24

**Table 3:** **Outpatient utilization of psycholeptics (ATC subgroup N05) in the City of Zagreb in 2001 according to financial indicators (€)**

ATC code	Therapeutic subgroup	Generic drugs (€)	Brand name drugs (€)	Total (€)	Share of generic drugs%	Share of brand name drugs%
N05A	Antipsychotics	222 656.01	620 338.18	842 994.19	26.41	73.59
N05B	Anxiolytics	484 325.92	71 909.74	556 235.66	87.07	12.93
N05C	Hypnotics and sedatives	44 813.71	1601.97	46 415.68	96.55	3.45
N05	Psycholeptics	751 795.65	693 849.9	1 445 645.55	52.00	48.00

**Table 4:** **Outpatient utilization of psycholeptics (ATC subgroup N05) in the City of Zagreb in 2001 according to DDD/1000/day**

ATC code	Therapeutic subgroup	Generic drugs DDD/1000/day	Brand name drugs DDD/1000/day	Total DDD/1000/day	Share of generics%, DDD/1000/day	Share of brand name drugs% DDD/1000/day
N05A	Antipsychotics	10.49	5.04	15.53	67.54	32.45
N05B	Anxiolytics	59.5	10.25	69.75	85.31	14.69
N05C	Hypnotics and sedatives	8.45	0.1	8.55	98.82	1.18
N05	Psycholeptics	78.44	15.38	93.82	83.6	16.4

**Table 5:** **Outpatient utilization of psychopharmaceuticals (ATC subgroup N03-N06) in the City of Zagreb in 2010 according to financial indicators (€)**

ATC code	Therapeutic subgroup	Generic drugs (€)	Brand name drugs (€))	Total (€)	Share of generic drugs%	Share of brand name drugs%
N03AE-AX	Antiepileptics	205 294.17	154 448.51	359 742.68	57.07	42.93
N04 AA	Anticholinergics (biperiden)	112.79	11 656.96	11 769.75	0.96	99.04
N05	Psycholeptics	760 537.41	911 429.43	1 671 966.84	45.49	54.51
N06A	Antidepressants	152 064.2	328 916.08	480 980.28	31.62	68.38
Total		1 118 008.58	1 406 451.00	2 524 459.58	44.29	55.71

**Table 6:** **Outpatient utilization of psychopharmaceuticals (ATC subgroup N03-N06) in the City of Zagreb in 2010 according to DDD/1000/day**

ATC code	Therapeutic subgroup	Generic drugs DDD/1000/day	Brand name DDD/1000/day	Total DDD/1000/day	Share of generics% DDD/1000/day	Share of brand name drugs% DDD/1000/day
N03AE-AX	Antiepileptics	3.39	3.21	6.49	51.36	49.46
N04 AA	Anticholinergics (biperiden)	0.01	0.9	0.91	1.1	98.9
N05	Psycholeptics	62.54	13.71	76.25	82.02	17.98
N06A	Antidepressants	8.17	12.34	20.51	39.83	60.17
Total		74.11	30.16	104.27	71.08	28.92

**Table 7:** **Outpatient utilization of psycholeptics (ATC subgroup N05) in the City of Zagreb in 2010 according to financial indicators**

ATC code	Therapeutic subgroup	Generic drugs (€)	Brand name drugs (€)	Total (€)	Share of generic drugs%,	Share of brand name drugs%
N05A	Antipsychotics	292 507.98	728 406.31	1 020 914.29	28.65	71.35
N05B	Anxiolytics	367 389.77	155 872.08	523 261.85	70.21	29.79
N05C	Hypnotics and sedatives	100 639.65	5923.39	106 563.04	94.44	5.56
N05	Psycholeptics	760 537.41	911 429.43	1 671 966.84	46.26	53.74

**Table 8:** **Outpatient utilization of psycholeptics (ATC subgroup N05) in the City of Zagreb in 2010 according to DDD/1000/day**

ATC code	Therapeutic subgroup	Generic drugs DDD/1000/day	Brand name drugs DDD/1000/day	Total DDD/1000/day	Share of generics%, DDD/1000/day	Share of brand name drugs% DDD/1000/day
N05A	Antipsychotics	3.93	4.49	8.42	46.67	53.33
N05B	Anxiolytics	43.43	8.88	52.31	83.02	16.98
N05C	Hypnotics and sedatives	15.18	0.34	15.52	97.81	2.19
N05	Psycholeptics	62.54	13.71	76.25	82.02	17.98

**Table 9:** **Comparison of outpatient utilization of generic drugs from the main ATC group N (N03-N06) in the City of Zagreb in 2001** ***versus*** **2010 – generic drug/brand name drug ratio**

ATC code	Financial indicators		DDD/TID	
	Ratio generics/originators	Ratio generics/originators	Ratio generics/originators	Ratio generics/originators
	2001	2010	2001	2010
N03AE-AX	0.84	1.33	3.08	1.04
N04 AA	0.00	0.01	0.00	0.01
N05	1.08	0.83	5.10	4.56
N06A	0.91	0.46	2.82	0.66
Total	0.98	0.80	4.20	2.46

In 2001, the share of generic psychopharmaceuticals in total drug utilization was 49.55% and 80.76% according to financial indicators and DDD/1000/day, respectively (Tables 1 and 2).

Psycholeptics (ATC subgroup N05) accounted for the highest share of generic *versus* brand name psychopharmaceuticals according to both financial indicators (52%) and DDD/1000/day (83.6%). In the N04A therapeutic subgroup, biperiden was the only agent observed, which was included in the Croatian Institute of Health Insurance (CIHI) List of Drugs only as brand name drug, thus precluding consumption of the generic agent.

Outpatient utilization of psycholeptics in the City of Zagreb in 2001, expressed according to financial indicators and DDD/1000/day is presented in Tables 3 and 4.

The highest consumption of generic drugs was recorded in the therapeutic subgroup of hypnotics and sedatives (96.55% according to financial indicators and 98.82% according to DDD/1000/day) and lowest consumption in the therapeutic subgroup of antipsychotics (26.41% and 67.57%, respectively).

Outpatient utilization of psychopharmaceuticals (ATC subgroup N03-N06) in the City of Zagreb in 2010, expressed according to financial indicators and DDD/1000/day, is illustrated in Tables 5 and 6.

Outpatient utilization of psycholeptics in the City of Zagreb in 2010, expressed according to financial indicators and DDD/1000/day is presented in Tables 7 and 8.

The share of generic psychopharmaceuticals in total drug utilization was 44.78% according to financial indicators and 71.08% according to DDD/1000/day. The highest consumption was recorded for psycholeptics (ATC subgroup N05) 46.26% according to financial indicators and 82.02% according to DDD/1000/day) and lowest for biperiden (N04A) (0.96% and 1.1%, respectively). These were followed by the consumption of generic antidepressants, which accounted for 31.62% according to financial indicators and 39.83% according to DDD/1000/day as compared with brand name agents.

In 2010, the highest consumption of generic drugs in the N05 subgroup was recorded in the therapeutic subgroup of hypnotics and sedatives (94.44% according to financial indicators and 97.81% according to DDD/1000/day) and lowest consumption in the therapeutic subgroup of antipsychotics (28.65% and 46.67%, respectively).

During the period of observation, the share of generic psychopharmaceuticals in total drug utilization decreased by 9.6% according to financial indicators and by 12% according to DDD/1000/day. The greatest decline was recorded in the therapeutic subgroup of antidepressants, i.e. by 33.8% according to financial indicators and by 46% according to DDD/1000/day. In the therapeutic subgroup of antiepileptics (N03AE-AX), the share of generic drugs in total drug utilization decreased by 32% according to DDD/1000/day, but increased by 25.1% according to financial indicators.

The ratio of generic and brand name psychopharmaceuticals (ATC subgroup N03-N06) in the City of Zagreb in 2001 and 2010 is shown in Table 9. The highest generic/brand name drug ratio according to DDD/1000/day was recorded for the group of psycholeptics and lowest for biperiden and the group of antidepressants. In comparison with 2001, the ratio showed reduction for all groups individually and for all psychopharmaceuticals taken together.

## Discussion

One of the ways to rationalize drug utilization and upgrade the quality of drug prescribing is promotion of prescribing generic drugs. According to the WHO (EURO-MED-STAT Group), drug utilization can be considered rational if the ratio of generic and brand name drug costs is around 80% in favor of generic drugs [18].

Results of the present study revealed the cost of psychopharmaceuticals in the City of Zagreb to have increased by 20.1% during the study period, with a significantly greater increase recorded for brand name drugs as compared with generic drugs (32.7% *vs*. 7.4%).

Differences were found in the distribution and trends in the outpatient utilization of generic psychopharmaceuticals according to both DDD/1000/day and financial indicators. The rate of prescribing generic psychopharmaceuticals was lower in 2010 than in 2001 according to both parameters. In 2001, the share of generic psychopharmaceuticals in total drug utilization according to DDD/1000/day was 80.76%, which corresponded with the criterion of rational consumption. During the study period, however, the share of generic psychopharmaceuticals in total drug utilization according to DDD/1000/day declined by 12%, accounting for 71.08% in 2010, although the number of generic drugs included in the CIHI List of Drugs had increased. From 2001 to 2010, many changes were made in the CIHI List of Drugs, which also influenced drug utilization.

Total cost of antidepressants increased from 2001 to 2010 by 32.6%, whereby the cost of generic drugs declined by 12.3%, while the cost of brand name drugs increased b 73.6%. The greatest decline in the share of generic drugs in total utilization was recorded in this therapeutic subgroup, i.e. by 33.8% and 46% according to financial indicators and DDD/1000/day, respectively. In the therapeutic subgroup of antidepressants included in the CIHI List of Drugs, the choice to prescribe a generic or an brand name drug was only possible for fluoxetine [10], whereas other agents were either generic (amitriptyline, clomipramine and maprotiline) or brand name (paroxetine, sertraline, fluvoxamine, tianeptine and reboxetine). According to DDD/1000/day, consumption of the generic agent fluoxetine significantly exceeded consumption of the brand name drug (4.06:0.22).

During the study period, new antidepressants were included in the List, as follows: moclobemide (1 brand name), citalopram (2 generics), escitalopram (1 brand name and 2 generics), venlafaxine (4 generics), mirtazapine (2 generics) and duloxetine (1 brand name). At the same time, the number of generic antidepressants previously included in the List increased, as follows: fluoxetine (1 generic), paroxetine (3 generics) and sertraline (5 generics). Fluvoxamine and tianeptine have now been transferred to the Supplementary list of Drugs with patient co-payment.

In 2010, the CIHI List of Drugs offered choice on prescribing generic or brand name antidepressant for fluoxetine, escitalopram (supplementary list), paroxetine and sertraline [19, 20]. Although the share of generic fluoxetine according to DDD/1000/day exceeded the share of brand name drug (1.18:0.58), it was significantly lower according to financial indicators. There was no utilization of generic escitalopram, while consumption of the brand name drug was 2.34 DDD/1000/day. According to this indicator, the utilization of generic paroxetine and generic sertraline was significantly lower in comparison with the brand name drugs (0.46:3.3 and 0.87:3.58, respectively).

Although a generic drug is therapeutic equivalent to the brand name one and is of the same quality, safety and efficacy as the brand name drug, the higher rate of prescribing brand name, more expensive drugs relies on the studies suggesting that even minimal variation in drug bioequivalence and efficacy may play a major role due to the complex nature of the disease [21, 22]. The more so, a study comparing the economic effect of substituting brand name selective serotonin reuptake inhibitors (SSRIs) with generic SSRIs in patients with major depressive disorder pointed to the possible increase in the overall cost of treatment due to the increased rate of hospitalization, emergency visits to physician offices and other associated costs caused by therapy noncompliance [23]. In contrast, other studies found no statistically significant difference in therapy compliance between patients treated with generic and brand name SSRI/serotonin-norepinephrine reuptake inhibitor (SNRI) antidepressants, or between those on generic and brand name escitalopram [24, 25]. Some authors suggest restrictions in prescribing brand name and newer antidepressants in order to increase the use of generic antidepressants, as a stimulating measure to modify prescribing habits [4]. Prescribing restrictions have been successfully introduced in Austria to limit the prescribing of atorvastatin and rosuvastatin *versus* generic statins. Specific measures in the case of antidepressants could include academic detailing, prescribing guidance, and prescribing restrictions to encourage the prescribing of multiple-sourced antidepressants such as venlafaxine and mirtazapine where appropriate. The restricions would limit the prescribing duloxetine to second line after mirtazapine, or venlafaxine if there are concerns with their side effects or effectiveness in practice [4]. In Sweden, prescribing duloxetine is restricted, with due consideration of individual side effects and efficacy in practice [4]. The utilization of venlafaxine significantly increased after prescription restrictions for duloxetine [26].

In the chemical-therapeutic subgroup of mood stabilizers (N03AE-AX), total cost increased by 2.1%. The share of generic drugs decreased according to DDD/1000/day, but increased according to financial indicators. In 2001, the CIHI List of Drugs included either generic or brand name mood stabilizers (clonazepam, brand name; carbamazepine and Na-valproate, generics). The increasing number of generic drugs on the CIHI List of Drugs was accompanied by the growing number of brand name drugs. Until 2010, the following new drugs were introduced: gabapentin (1 brand name and 2 generics), oxcarbazepine (1 brand name), pregabalin (1 brand name) and levetiracetam (1 brand name). In addition, new lamotrigine (6 generics) and topiramate (2 generics) drugs were included.

In 2010, there was choice between generic and brand name lamotrigine and gabapentin. Clonazepam, oxcarbazepine, pregabalin and levetiracetam were only available as brand name drugs, and carbamazepine, Na-valproate and topiramate as generic drugs [10, 19]. The utilization of generic lamotrigine according to DDD/1000/day was significantly lower as compared with the brand name drug (0.27:1.0), whereas the utilization of generic and brand name gabapentin was quite comparable (0.10:0.13). Although physicians may want to consider more intensive monitoring of high-risk patients taking antiepileptic drugs when any medication change occurs, in the absence of better data, there is little evidence-based rationale to challenge the implementation of generic substitution for antiepileptic drugs in most cases [27]. Results of some other studies that compared the use of generic and brand name lamotrigine in patients with epilepsy and bipolar disorder also report no statistically significant difference either according to plasma drug concentration or exacerbation of the disease main symptoms [28, 29].

During the study period, the highest share of generic *versus* brand name drug utilization was recorded for psycholeptics (N05) according to both financial indicators and DDD/1000/day, in spite of the respective decline by 11% and 1.9%. Total cost of hypnotics and sedatives increased by 130%, however, significantly more for brand name (270%) than for generic agents (124%). In the subgroup of hypnotics and sedatives, either generic (flurazepam, nitrazepam, zolpidem) or brand name (midazolam) agents were included in the CIHI List of Drugs in 2001 [10]. Among generic drugs, nitrazepam showed highest utilization (6.24 DDD/1000/day), followed by zolpidem (1.74 DDD/1000/day), and flurazepam (0.46 DDD/1000/day). The use of midazolam (brand name drug) was only 0.1 DDD/1000/day. In the study period, a new hypnotic zaleplon (1 generic) and a new zolpidem (1 generic) were added to the Supplementary list [20].

In 2010, physicians could choose prescribing either generic or brand name hypnotic and sedative, although a greater number of generic drugs were available as compared with 2001 [19].

The options included generic flurazepam, nitrazepam and zolpidem on the main list *versus* more expensive zaleplon on the supplementary list [19, 20]. Of brand name agents, only midazolam was available. Among generic agents, zolpidem was most commonly prescribed (12.57 DDD/1000/day), so its consumption increased considerably from 2001; it was followed by nitrazepam (2.2 DDD/1000/ay), flurazepam (0.4 DDD/1000/day) and zaleplon (0.05 DDD/1000/day) from the supplementary list. However, utilization of the brand name drug midazolam increased to 0.33 DDD/1000/day in 2010.

In the therapeutic subgroup of antipsychotics (N05A), the share of generic drugs decreased significantly from 2001, by 30.9% according to DDD/1000/day, but increased by 8.5% according to financial indicators. Total cost of antipsychotics also increased by 21.1% (brand name drugs by 17.4% and generic drugs by 31%). In 2001, either generic or brand name drugs were included in the CIHI List of Drugs. Of typical antipsychotics, the brand name agents levopromazine, perazine and thioridazine, or generic agents promazine and fluphenazine were prescribed. Levopromazine, a typical antipsychotic included in the List in 2001 as a brand name drug was in the interim replaced by a generic drug.

Of atypical antipsychotics, the brand name agents (clozapine, olanzapine and risperidone) or generic agents (sulpiride) could be prescribed [10]. In 2010, the number of generic antipsychotics, both typical and atypical, included in the CIHI List of Drugs increased since a number of new atypical antipsychotics were added to the List in the study period: quetiapine (2 brand name and 3 generics), ziprasidone (1 brand name and 1 generic), sertindole (1 brand name), zuclopentixol (2 brand name) and amisulpride (1 brand name). At the same time, the number of formulations of the antipsychotics previously present on the List increased, as follows: promazine (1 generic), clozapine (1 brand name), olanzapine (1 brand name and 5 generics) and risperidone (5 generics) [19].

Concerning typical antipsychotics, only generic drugs could be prescribed in 2010. Of atypical antipsychotics, the following generic drugs could be prescribed: ziprasidone, clozapine, olanzapine, quetiapine, sulpiride and risperidone. The following brand name agents were included in the CIHI List of Drugs: sertindole, zuclopentixol and amisulpride. Among atypical antipsychotics, there was choice between generic and brand name ziprasidone, clozapine, olanzapine, quetiapine and risperidone [19]. According to DDD/1000/day, the utilization of the generic olanzapine was significantly lower as compared with the brand name drug (0.58:2.2), while the respective ratio was lower in case of risperidone (0.32:0.52). While there was no utilization of the generic ziprasidone, clozapine and quetiapine, their respective brand name drugs were used as follows: ziprasidone 0.22, clozapine 0.7 and quetiapine 0.68 DDD/1000/day.

The declining trend recorded in the utilization of generic antipsychotics could in part be attributed to the decreasing use of typical antipsychotics (only generic agents included in the CIHI List of Drugs in 2010) and the increasing use of atypical antipsychotics, where ever more generic and brand name drugs were available, and physicians tended to more frequently prescribe the more expensive brand name ones. Considering quite frequent therapy noncompliance in schizophrenics (40%-50% according to some authors), which can, among others, be due to therapy related factors, physicians are probably reluctant to prescribe generic drugs [30, 31]. Depression, schizophrenia and bipolar disorder are very complex disorders; therefore physicians avoid switching their therapy, especially if the patient’s state remains stable on particular medication. Based on the German pharmacoeconomic models, it is concluded that the effects of the brand name risperidone substitution with generic drug are not cost effective if resulting in reduced patient compliance. The lack of compliance leads to poorer disease control, which in turn has unfavorable effects on the patient’s quality of life, increases the rate of hospitalization, and eventually increases the overall cost of treatment [32]. Similar results have also been reported for generic *versus* brand name clozapine [33]. However, this has not been the case with more recent formulations of generic clozapine, or especially generic olanzapine. Relapse rates were similar in patients before and after switching to generic olanzapine, and no untoward side effects were seen in any patient prescribed generic olanzapine [34].

Trend in the utilization of the anticholinergic biperiden used in the management of these disorders can be taken as an indirect indicator of the psychopharmaceutical prescribing quality, i.e. a reduced rate of extrapyramidal side effects [35]. Although total consumption of biperiden decreased during the study period, the share of generic biperiden in total utilization increased due to the fact that only the brand name drug was included in the CIHI List of Drugs in 2001, whereas generic biperiden was added along with the brand name drug in 2010 [10, 19].

In 2010, a decline was recorded in the utilization of generic anxiolytics relative to total anxiolytic utilization, by 19.4% according to financial indicators and by 2.7% according to DDD/1000/day. At the same time, a greater number of generic anxiolytics were included in the CIHI List of Drugs. New generic formulations of alprazolam were introduced, i.e. 1 on the essential list and 2 on supplementary list. Considering all psychopharmaceuticals, total cost decreased only for anxiolytics (by 6%), although the cost increased for brand name drugs and decreased for generic drugs. In 2001, the generic drugs diazepam, oxazepam, lorazepam and meprobamate were included in the CIHI List of Drugs, while physicians could choose between generic and brand name alprazolam. In 2010, they could choose between generic and brand name diazepam and alprazolam, and generic oxazepam and lorazepam. Diazepam and alprazolam were included in the essential list, while oxazepam, lorazepam and alprazolam were included in the supplementary list [19, 20].

The utilization of generic diazepam was tenfold the utilization of brand name diazepam (16.26:1.66 DDD/1000/day), while the utilization of generic alprazolam was twofold the utilization of brand name alprazolam (8.25:6.68 DDD/1000/day).

Many industrialized countries have enacted an array of concrete measures aimed at rationalization of drug utilization, such as defining the price of generic products, stimulating pharmacists to issue generic products, and giving priority to generic products on the lists of insurance companies [4, 8, 9, 36, 37]. In Austria, the price of brand name and generic drugs is regulated so that the price of a third generic must be at least 60% below the single-sourced prices to be reimbursed, which stimulated the market to lower prices, for successive branded generics in particular [4]. Lithuania has demonstrated that even small countries can have low prices of generic drugs [38].

In Croatia, drug prescribing is characterized by continuous efforts to use the restrictive financial resources for drugs in the most rational way. With this in mind, various administrative measures were launched on several occasions, but of limited duration, such as a limited number of prescriptions *per* patient *per* year [39].

During 2009 and 2010, drug pricing and reimbursement were also revised as part of the health reform, in order to improve the value for money and decision making efficacy, while at the same time enabling the practice of ethical drug promotion [16]. The following measures were most relevant:

1. The first generic available will have the price set 30% below the originator and each subsequent generic will be 10% below the previous generic on the Croatian positive list [8].
2. If a medicine is considered eligible for reimbursement, it will be put on one of two positive lists: List A, essential list providing 100% reimbursement of the reference price for listed products, and List B, supplementary list where patients are charged co-payments [8].
3. A reference price system is a pharmaceutical reimbursement element in which identical or similar products are clustered in so-called reference groups. Reference prices are determined on the basis of the lowest price of a product which recorded at least 5% of sales within a therapeutic group over a twelve-month period to avoid the possibility of market shortages [8].

A key element in the Croatian reimbursement system is reference price system, while generic substitution is neither mandatory nor motivated by a financial incentive that could serve as a positive factor for increasing generic uptake. In Croatia, incentives for generic promotion are not considered necessary since the Social Insurance pays the reference prices, and as a consequence most manufacturers lower their prices to avoid co-payments [8].

Although the demand-side measures have been successfully implemented in Croatia, having resulted in improved renin-angiotensin prescribing efficiency [9], it does not hold for psychopharmaceuticals. The measures undertaken for rationalization of psychopharmaceutical utilization in Zagreb and Croatia during the study period failed to fulfill the set goal completely. The main reason for this failure lies in the fact that the measures undertaken in Croatia generally referred to drug pricing, including lowering the price of generic drugs, while numerous other demand-side measures counterpoising commercial pressures and encouraging prescribing generic drugs failed to take place. Similar to this, in Scotland and Belgium, the introduction of generic risperidone as the sole measure did not increase the overall utilization of risperidone because it was not accompanied by the specific additional measures to stimulate its utilization [36, 40, 41].

In Croatia, the lack of uniform national guidelines and the still great impact of pharmaceutical industry marketing continue favoring the ever increasing prescribing brand name drugs, thus making the overall drug cost ever more expensive. More specific findings include the fact that both suply and demand-side reforms are essential to maximize prescribing efficiency [6, 42]. Combining the initiatives to lower the price of generics with demand-side measures to enhance their prescribing is important to maximize prescribing efficiency. Just addressing one component will limit the potential efficiency gains. Demand-side measures based on the four “E”s include educational activities, engineering activities, economic intervention and enforcement [6].

There is a potential to achieve some savings with generic atypical anitpsychotic drugs. However, this is limited by the complexity of the disease area, so multiple measures are needed to change the physician prescribing habits. Authorities cannot rely on any ‘spillover’ effects to affect future prescribing, even in closely related classes [36, 40, 41].

One of the limitatations of this study was the limitation of the DDD methodology itself, as the defined daily dose does not necessarily reflect the recommended or prescribed daily dose. This methodology provides only a rough estimate of consumption and not an exact picture of actual use. Data on the diagnoses for which the drugs were prescribed would also have been useful in further study.

## Conclusions

During the ten-year period of observation, a decrease in the share of generic psychopharmaceuticals in total drug utilization was recorded by both DDD/1000/day and by financial indicators, in spite of the fact that the number of generic drugs included in the CIHI List of Drugs increased.

The greatest decline was recorded in the therapeutic subgroup of antidepressants (46%), followed by mood stabilizers (32%) and antipsychotics (30.9%), while the least decrease was found in the subgroup of hypnotics and sedatives (1%).

Total cost of psychopharmaceuticals increased by 20.1%, 32.7% for brand name and 7.4% for generic agents. Total cost of antidepressants increased by 32.6%, whereby the cost of generic drugs decreased by 12.3% and the cost of brand name drugs increased by 73.6%. Total cost also increased for the subgroups of hypnotics and sedatives (130%) and of antipsychotics (21.1%), while a decrease was only recorded for the subgroup of anxiolytics (6%).

Although mental disorders are very complex diseases and physicians are reluctant to switch therapy, there is still room for additional savings and more rational utilization of psychopharmaceuticals through stimulating the use of generic drugs, along with individualized therapy protocols.

To achieve this goal, additional, specific measures are needed to stimulate prescribing generic antidepressants and antipsychotics. These measures should include target education on the quality, safety and efficacy of generic drugs, national guidelines, transparent method of generic drug pricing, defining the criteria for adding a generic product on the CIHI List of Drugs, quality control of generic products on the market, and ensuring supply with the generic drugs included in the CIHI List of Drugs. Restrictive prescribing measures can also prove useful.
